# Effect of knee arthroscopic debridement combined with peripatellar denervation on restoration of knee function in patients with knee osteoarthritis

**DOI:** 10.1186/s12893-023-02113-4

**Published:** 2023-08-11

**Authors:** Zhijia Wang, Rui Wang, Congliang Gao

**Affiliations:** Department of Bone Trauma and Arthrology, Huai’an Hospital, No. 19, Shanyang Avenue, Huai’an District, Huai’an, 223200 Jiangsu Province China

**Keywords:** Knee arthroscopy, Joint debridement, Peripatellar denervation, Knee osteoarthritis, Knee function

## Abstract

**Background:**

This research examines knee osteoarthritis (OA), a prevalent orthopedic disease marked by cartilage degeneration and chronic synovitis, leading to pain, restricted mobility, and eventual loss of knee function. Notably, patellofemoral osteoarthritis constitutes a significant proportion of knee OA cases. Our study aims to assess the impact of knee arthroscopic debridement coupled with peripatellar denervation on restoring knee function in OA patients and analyze the risk factors affecting treatment outcomes. By doing so, we hope to contribute to the informed selection of clinical treatment plans, addressing a disease that, if untreated, significantly impairs patients’ quality of life.

**Methods:**

A total of 211 patients with knee osteoarthritis treated in our hospital from June 2020 to June 2022 were analyzed retrospectively. Among them, 116 patients received arthroscopic knee debridement treatment alone as the control group, and 95 in the observation group were combined with denervation treatment based on the control group. The clinical efficacy of the two groups of patients after treatment was evaluated, and patients’ pain was counted using the pain visual analogue score (VAS) method. The knee range of motion (ROM) was used to count the mobility of the patients and to compare the operative time, intraoperative perfusion volume, and length of stay between the two groups. According to the effectiveness after treatment, patients were divided into the improvement group (effective + markedly effective) and the non-improvement group, and the risk factors affecting the clinical efficacy of patients after treatment were analyzed by logistic regression.

**Results:**

The total treatment efficiency of patients in the control group was lower than that of those in the observation group (P < 0.05). There was no difference in intraoperative perfusion volume and length of stay between patients in both groups (P > 0.05). However, the operative time was shorter in the control group compared with that in the observation group (P < 0.001). The post-treatment VAS scores of patients in the observation group were lower than those in the control group, while the ROM scores were higher than those of the control group (P < 0.001). Age, BMI, and preoperative VAS score were found to be independent risk factors for patient outcome by logistic regression analysis (P < 0.05).

**Conclusion:**

knee arthroscopic debridement combined with peripatellar denervation has a significant improvement in the restoration of knee function in patients with knee osteoarthritis and reduces their level of pain.

## Introduction

Knee osteoarthritis is a common orthopedic disease characterized by degenerative changes in the cartilage and chronic synovitis, which is a long-term chronic progression of a combination of factors, accompanied by pain and limitation of movement, leading to further loss of knee function [1, 2]. Among knee osteoarthritis, patellofemoral osteoarthritis is more common and has a larger proportion and is also a common disease in orthopedic clinics [3]. In one statistic, isolated patellofemoral arthritis was found in about 67% of patients with symptoms of knee osteoarthritis and was more common in women aged 40–50 years [4]. Isolated patellofemoral arthritis is present in approximately 2% of middle-aged and older men over 55 years of age, and the incidence of pure patellofemoral arthritis is approximately 9% in those over 40 years of age with knee pain [5]. Once osteoarthritis has progressed to an advanced stage, the significant cost of treatment will place a huge burden on the national community.

It was found that the incidence of anterior knee pain increases gradually with age. If treatment is not timely, it will accelerate the degeneration of knee cartilage, which will lead to the loss of joint function of the knee, resulting in a significant decrease in the quality of life of patients. When patients’ symptoms are not relieved or aggravated after early conservative treatment, patients are recommended to undergo surgery [6, 7]. Currently, the main clinical treatment options regarding knee osteoarthritis include arthroscopic surgery, knee replacement and knee fusion [8]. Knee arthroscopy, as a minimally invasive procedure, can effectively slow down the progression of the disease [9]. However, some scholars believe that the treatment of arthroscopic joint debridement of the knee is no different from placebo surgery [10]. Peripatellar denervation has an anatomical mechanism for reducing patellofemoral joint pain, and peripatellar cartilage is cauterized around the patellar plexus with a radiofrequency ion knife without causing damage to the anterior peripatellar tissues. The patellar trophoblastic vessels are mainly in the anterior and superior regions of the patella, and peripatellar denervation does not block peripatellar blood flow leading to complications such as patellar fracture and necrosis [11]. Whether the combination of the two treatments improves patient outcomes has not been demonstrated in studies, and the risk factors for clinical outcomes remain unclear.

In this study, we aimed to analyze the effect of knee arthroscopic debridement combined with peripatellar denervation on the restoration of knee function in patients with knee osteoarthritis, and to analyze the risk factors affecting patients’ treatment outcome, so as to provide reference for the selection of clinical treatment plan.

## Materials and methods

### Clinical data

A total of 211 patients with knee osteoarthritis treated in our hospital from June 2020 to June 2022 were analyzed retrospectively. Among them, 116 patients received arthroscopic knee debridement treatment alone as the control group, and 95 in the observation group were combined with denervation treatment based on the control group. The study was conducted with the approval of our medical ethics committee.

### Inclusion and exclusion criteria

Inclusion criteria: Patients all met the diagnostic criteria for osteoarthritis of the knee established by the American Rheumatism Association [12]. The duration of pain symptoms was less than 1 year. All patients had the disease unilaterally. The patient had a confirmed diagnosis of knee osteoarthritis by X-ray and MRI. Patients were of Kellgren-Lawrence grade at grades II-IV. Patients’ clinical data were complete.

Exclusion criteria: anterior and posterior cruciate ligament, meniscus, and medial and lateral collateral ligament injuries; patients with significant lower extremity force line abnormalities such as knee deformities visible on X-rays; patients with concomitant knee infections; those with severe heart, lung, brain and blood vessel diseases; patients with imaging manifestations suggestive of patellar tilt, subluxation, and synovial crepitus; patients with combined malignancy; Patients with bilateral dysfunction were excluded. The patient had not undergone a corresponding surgical treatment prior to this study.

### Treatment options

The control group was treated with knee arthroscopic debridement, with the patient in the supine position, for which intralesional anesthesia was administered. After routine disinfection and spreading of the towel, an incision was made on the anteromedial side of the affected knee and the arthroscope was inserted. Under arthroscopic guidance, the joint was first routinely lavaged and the free body was removed. Most of the synovial membrane of the hyperplasia was shaved, and the hyperplastic bone was ground and removed using a grinding drill, and the burrs and hyperplastic tissue on the surface of the damaged cartilage and degenerated articular cartilage were carefully cleaned. To promote adequate flow of internal lavage fluid in patients with severe articular cartilage metaplasia, it is also necessary to use a microfractor to punch holes in the cartilage defect area from the outside inwards, and to loosen it if necessary. After completing these operations, the joint cavity is repeatedly flushed with saline in order to remove the dislodged free material and debris completely. Finally, negative pressure drainage is placed in the suprapatellar capsule.

The observation group received knee arthroscopic debridement in combination with denervation treatment. The arthroscopic debridement procedure was the same as that of the control group. For the denervation treatment, the peripatellar nerve was denervated and cauterized with microscopic radiofrequency for approximately 5 to 10 cm prior to drilling and microfracture. Additionally, the joint capsule, as well as the anterior, posterior, and lateral aspects of the patellar epicondyle, were cauterized using an electric knife to ensure thorough cleaning of the joint cavity. Finally, the incision was closed layer by layer. Postoperative denervation did not cause related complications and related index changes.

### Outcome measures

Main outcome measures: The clinical outcomes of the two groups of patients after treatment were evaluated on the following criteria: Markedly effective: After treatment, patients’ knee pain and swelling disappeared completely, and the joint movement and function returned to normal. Effective: After treatment, patients’ knee pain and swelling symptoms occurred occasionally, and joint mobility was enhanced. Ineffective: After treatment, patients’ knee pain and swelling symptoms did not improve, and joint movement was still limited. (Effective + Markedly effective) / Total number of cases × 100% = Total effective rate. The pain visual analogue score (VAS) [13] method was used to count patients’ pain before and after 3 months of treatment, with a total score of 10, with lower scores indicating less joint pain. The knee range of motion (ROM) [14] was used to count patients’ pre-treatment and post-treatment mobility with a total score of 120, with higher scores representing higher knee mobility.

Secondary outcome measures: The differences in baseline data between the two groups of patients were compared. The operative time, intraoperative perfusion volume, and length of stay between the two groups were compared. According to the effective status of patients after treatment, patients were divided into the improvement group (effective + markedly effective) and the non-improvement group, and the risk factors affecting the clinical efficacy of patients after treatment were analyzed by logistic regression.

### Statistical analysis

The data collected in this study were statistically analyzed using the SPSS 20.0 package and the required images were drawn using the GraphPad 7 package. Data were expressed using mean ± standard deviation (Meas ± SD), and comparisons between groups were analyzed using independent samples t-test and within groups using paired t-test. The counting data were expressed as rate (%) and assessed using the chi-square test, expressed as χ^2^. Independent risk factors affecting the clinical outcome of patients were analyzed using logistic regression. The value of independent risk factors in predicting clinical outcomes of patients was analyzed using receiver operating characteristic curve (ROC). P < 0.05 indicates a difference.

## Results

### Comparison of clinical data

It was found that there was no statistical difference between the clinical data of patients in the two groups (Table 1, P > 0.05).

**Table 1:** Baseline data

Factor		Control group (n = 116)	Observation group (n = 95)	χ^2^ value	P value	
Gender				0.021	0.884	
	Male	50	40			
	Female	66	55			
Age				1.068	0.301	
	≥ 65 years	42	28			
	<65 years	74	67			
Course of disease				0.296	0.585	
	≥ 3 years	69	60			
	<3 years	47	35			
BMI				0.379	0.537	
	≥ 25 kg/m^2^	44	40			
	<25 kg/m^2^	72	55			
Affected side				0.396	0.529	
	Left side	60	45			
	Right side	56	50			
Past medical history						
	Hypertension	44	37	0.022	0.880	
	Diabetes	27	18	0.583	0.445	
Outerbridge Classification						
	II	32	32	1.116	0.572	
	III	55	39			
	IV	29	24			

Note: Body Mass Index (BMI). All data were tested by chi-square test

### Clinical efficacy evaluation

The clinical efficacy of the two groups of patients was evaluated before and after treatment, and it was found that the total effective rate of treatment was lower in the control group than in the observation group (Table 2, P < 0.05). Moreover, there were no associated complications in both groups in the current study.

**Table 2:** Clinical efficacy evaluation

Group	Markedly effective	Effective	Ineffective	Total efficiency
Control group (n = 116)	60 (51.72%)	33 (28.45%)	23 (19.83%%)	93 (80.17%)
Observation group (n = 95)	66 (69.47%)	21 (22.11%)	8 (8.42%)	87 (91.58%)
χ^2^ value				5.422
P value				0.019

Note: All data were tested by chi-square test

### Comparison of general data

**Fig. 1:**
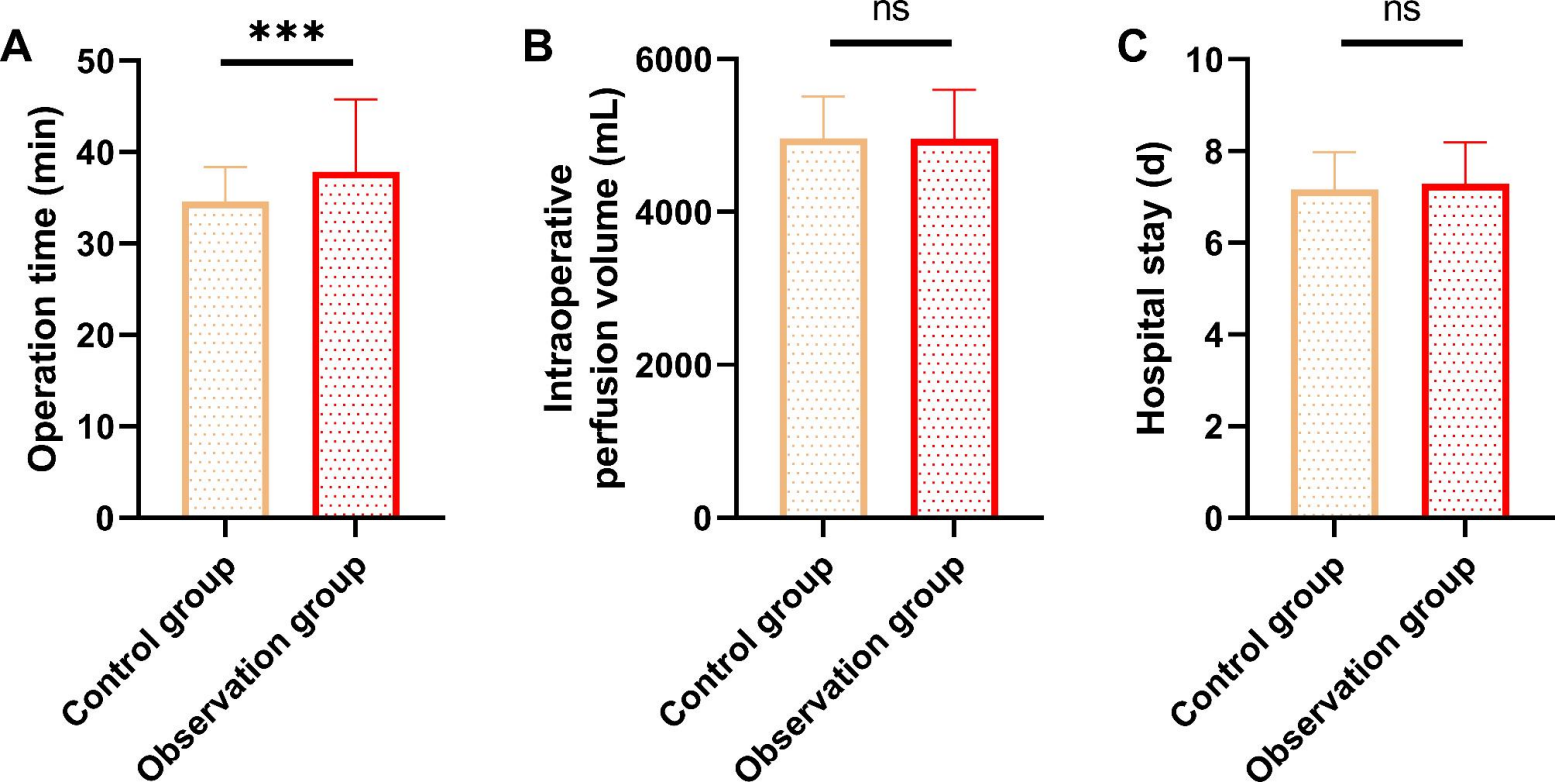
Comparison of general data of patients. **A.** Comparison of operative time between both groups of patients. **B.** Comparison of intraoperative perfusion volume between two groups of patients. **C.** Comparison of length of stay between two groups of patients Note: nsP > 0.05; ***P<0.001

A comparison of the general data of the two groups revealed that there was no difference in the intraoperative perfusion volume and hospital stay between both groups (Fig. 1A-B, P > 0.05), but the operative time was shorter in the control group than in the observation group (Fig. 1C, P < 0.001).

### Changes in pain conditions and activity

**Fig. 2:**
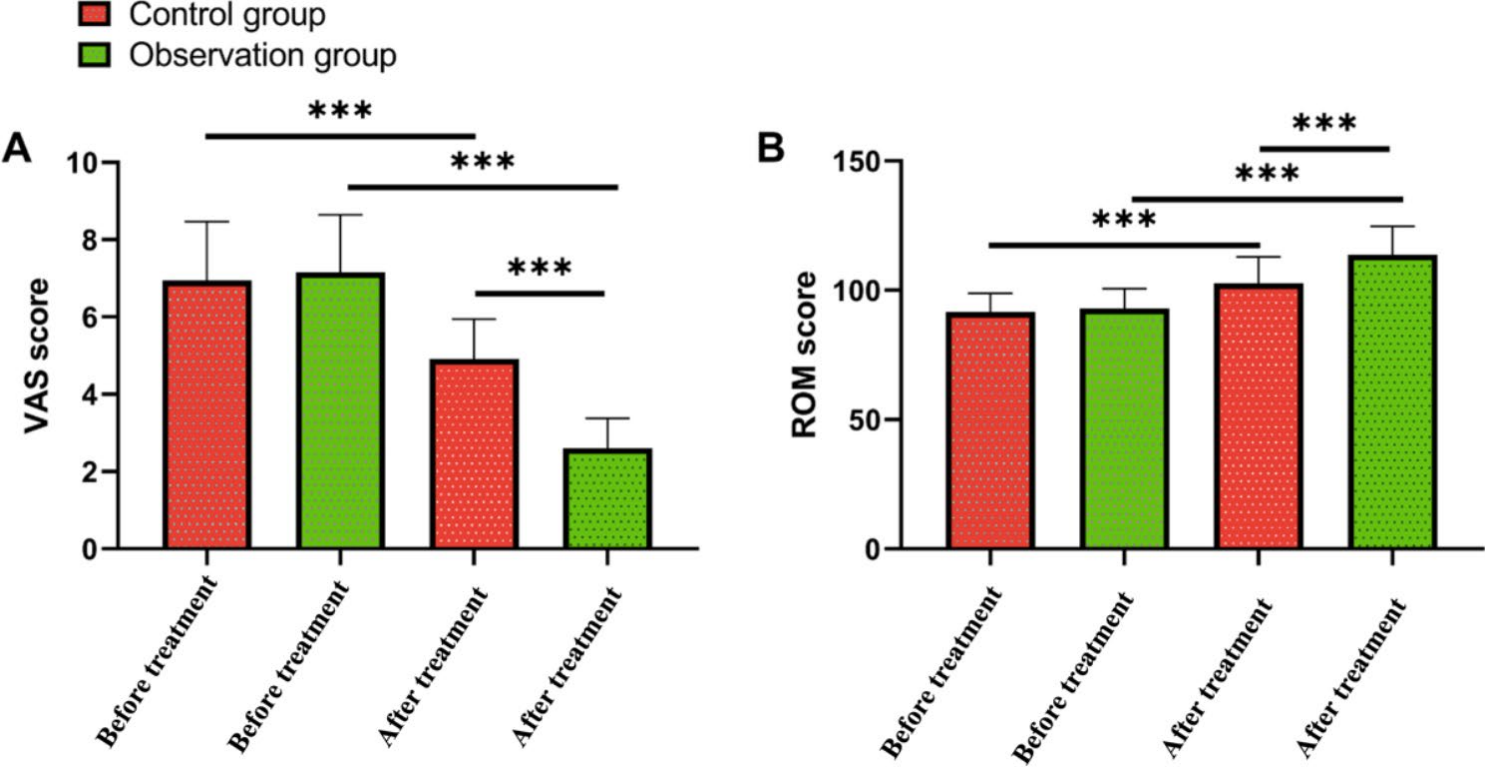
Changes in pain and activity of patients before and after treatment. **A.** Change in patients’ VAS scores before and after treatment. **B.** Change in patients’ ROM scores before and after treatment Note: ***P<0.001. Pain Visual Analogue Score (VAS); Knee Range of Motion (ROM)

The pain condition and activity of the two groups before and after treatment were evaluated. According to the results, the two groups were similar in VAS scores and ROM scores (Fig. 2, P > 0.05), while after treatment, both groups got notably decreased VAS scores and notably increased ROM scores (Fig. 2, P < 0.001). In addition, after treatment, the observation group got notably lower VAS scores and notably higher ROM scores than the control group (Fig. 2, P < 0.001).

### Analysis of risk factors affecting the efficacy of treatment

Patients were divided into improvement and non-improvement groups according to their clinical outcomes after treatment. Age, BMI, preoperative VAS score, and treatment regimen were found to be risk factors for patient outcomes by univariate analysis (Table 3, P < 0.05). We then assigned the data (Table 4). Age, BMI, and preoperative VAS score were found to be independent risk factors for patient outcomes by logistic regression analysis (Table 5, P < 0.05).

**Table 3:** Univariate analysis

Factor		Improvement group (n = 180)	Non-improvement group (n = 31)	χ^2^ value	P value
Gender				3.528	0.060
	Male	72	18		
	Female	108	13		
Age				23.411	<0.001
	≥ 65 years old	48	22		
	<65 years old	132	9		
Course of disease				0.143	0.704
	≥ 3 years old	113	22		
	<3 years old	67	15		
BMI				21.450	<0.001
	≥ 25 kg/m^2^	60	24		
	<25 kg/m^2^	120	7		
Affected side				0.890	0.345
	Left side	92	13		
	Right side	88	18		
Past medical history					
	Hypertension	63	18	0.704	0.401
	Diabetes	41	4	1.536	0.215
Operation time (min)		35.25 ± 5.59	36.21 ± 6.28	0.429	0.791
Intraoperative perfusion volume (mL)		4900.80 ± 616.95	4971.66 ± 585.36	0.113	0.909
Length of stay (d)		7.00 ± 0.96	7.25 ± 0.83	1.540	0.125
Preoperative ROM score		6.90 ± 1.49	7.89 ± 1.35	3.389	0.001
Preoperative VAS score		92.11 ± 7.63	92.51 ± 6.83	0.613	0.540

Note: Body Mass Index (BMI).

**Table 4:** Assignment table

Factor	Assignment
Age	≥ 65 years old = 1, <65 years old = 0
BMI	≥ 25 kg/m^2^ = 1, <25 kg/m^2^ = 0
Preoperative VAS score	Raw data are used for continuous variables
Treatment options	Control group = 1; observation group = 0
Efficacy	Improvement group = 0; non-improvement group = 1

**Table 5:** Logistics multivariate analysis

Factor	B value	Standard error	χ^2^	P value	Odds ratio	95% CI	
						Lower limit	Upper limit
Age	2.160	0.492	19.317	<0.001	8.675	3.310	22.735
BMI	2.008	0.515	15.193	<0.001	7.445	2.713	20.431
Preoperative VAS score	0.457	0.181	6.414	0.011	1.580	1.109	2.251
Treatment options	0.886	0.509	3.023	0.082	2.424	0.893	6.579

## Discussion

Knee osteoarthritis is a common chronic degenerative disease of osteoarticular cartilage, which can have a serious impact on the physical and mental health and quality of life of patients and needs to be treated as early as possible [15]. Anterior knee pain can be divided into meniscal-derived and non-meniscal-derived pain depending on the source, and meniscal-derived anterior knee pain can often be determined on the basis of pressure pain at the joint space and a positive McBurney sign, while patellofemoral disease is a common cause of non-meniscal-derived anterior knee pain [16].

An increasing number of studies have found that the pain caused by knee osteoarthritis is related to mechanical factors caused by damage to various tissues in the joint. Patients have a large amount of cartilage debris and a small number of free bodies in the knee cavity, which cause pain by impinging on the joint surface during activity, and the pain is also associated with the stimulation of nerve endings by various inflammatory nociceptive substances [17]. Knee arthroscopic debridement has advantages over other procedures such as knee arthrotomy and debridement in terms of less trauma, faster recovery, and repeatability [18]. Knee arthroscopic debridement can be performed to remove bone fragments that cause pain, wear out the internal structures of the knee joint, and affect the normal mobility of the joint, and to remove free bodies, effectively reducing pain of instrumental origin [18]. During the operation, the meniscus and joint surface are trimmed to reduce the irritation of the synovial membrane and to reduce the compression of the injured part on the joint movement. The contracted lateral support band explored during surgery was released to improve the abnormal anatomical and mechanical properties of the patellofemoral joint [19]. Although knee arthroscopic debridement can clean and remove the hyperplastic and congested inflammatory synovium and reduce the erosive effect of inflammatory factors on the knee joint, the efficacy is not ideal for patients with more severe pain. The peripatellar denervation treatment, by cauterizing the peripatellar and saphenous nerve branches with radiofrequency ion knife, can significantly decrease the number of injurious sensory neurons, thus reducing the release of neuropeptides and alleviating patients’ pain degree symptoms. At the same time, it can perform tightening treatment on the soft tissues around the joint surface to avoid the embeddedness of the surrounding soft tissues, thus reducing the pain symptoms [20]. In this study, we found that patients in the observation group had a significantly higher therapeutic efficiency than those in the control group after treatment. Moreover, patients in the observation group showed higher improvement in both VAS scores and ROM scores after treatment than those in the control group. This suggests that knee arthroscopic debridement combined with peripatellar denervation has a remarkable improvement in knee function and reduces pain levels in patients with knee osteoarthritis. Previously, Ou et al. [11] found that patients treated by peripatellar neurotomy in combination with cartilage cone and patellofemoral articular surface microfracture had significantly improved pain levels and enhanced knee function compared to those who underwent arthroscopic debridement alone. We believe that this is mainly due to the fact that radiofrequency cautery around the internal and external patellar condyles can effectively promote not only a significant reduction in patients’ neurogenic pain, but also improving the local microcirculation around patients’ knee joint, which in turn can effectively promote their knee joint mobility and functional recovery.

It has been shown that 50-80% of patients with osteoarthritis are in remission after arthrocentesis and can be maintained for one to five years [21]. However, there is no clarity regarding the risk factors that influence patient outcomes. In the present study, we analyzed the factors influencing the outcome of patients with knee osteoarthritis. In the current study, we found that age, BMI, and preoperative VAS score were independent risk factors for patient outcomes. The pathophysiological analysis of osteoarthritis is based on the degeneration of the hyaline cartilage of the articular surface with the exposure and sclerosis of the subchondral bone, which becomes more severe with the increase of patients’ age, and the hyaline cartilage in elderly patients has no regenerative capacity, which inevitably leads to a poor outcome after treatment [22]. The higher the body mass index, the more weight the patient bears on the knee joint, which increases exponentially when walking, running, and going up and down stairs, and the degree of osteoarthritis becomes more severe with increasing age and disease duration [23]. Sinan et al. [24] found that obese patients had poorer outcomes than patients with normal body mass and those with too little body mass. Creaby et al. [25] concluded that the risk of knee osteoarthritis increased by 20% every 5 years in patients with BMI ≥ 26, and the higher the BMI, the worse the efficacy of knee debridement. Pain is the most important reason that affects patients’ daily life and consultation, and it is a very precise symptom that patients feel and is an urgent problem that patients need to be solved [18]. The pain symptoms of patients are mainly related to the contact and collision between the articular surfaces. As the articular cartilage and subchondral bone are constantly destroyed in the process of pain, the more painful the joint is, the more serious the joint wear is, and the less the effect of arthroscopic debridement is. So, the higher the VAS score is, the worse the curative effect is.

In this study, we determined that knee arthroscopic debridement combined with peripatellar denervation had a significant improvement in knee function in patients with knee osteoarthritis. Nevertheless, the current study still has some limitations. For one thing, we cannot follow up the patients, and the controllability of rehabilitation training and other aspects after discharge was slightly poor. Whether the final surgical method varies in the recovery of knee function in patients with different grades is still unclear. For another, the postoperative follow-up time was short, and the long-term results of postoperative patients are yet to be observed. Thus, we hope to conduct more clinical trials in future studies to refine our findings. Besides, many new analytical methods may offer additional benefits to these patients [26, 27].

In conclusion, knee arthroscopic debridement combined with peripatellar denervation has a significant improvement in the restoration of knee function in patients with knee osteoarthritis and reduces their level of pain.

## Data Availability

All data generated or analyzed during this study are included in this published article.

## Abbreviations

VAS: Visual analogue score
ROM: Range of motion
ROC: Receiver operating characteristic curve
BMI: Body mass index
